# MST1 deletion protects β-cells in a mouse model of diabetes

**DOI:** 10.1038/s41387-022-00186-3

**Published:** 2022-02-08

**Authors:** Amin Ardestani, Kathrin Maedler

**Affiliations:** 1 Centre for Biomolecular Interactions Bremen, University of Bremen, Bremen, Germany; 2 Department of Molecular Medicine, School of Advanced Technologies in Medicine, Tehran University of Medical Sciences, Tehran, Iran

**Keywords:** Diabetes, Diabetes

## Abstract

The pro-apoptotic kinase Mammalian Sterile 20-like kinase 1 (MST1), an integral component of the Hippo pathway, is a key regulator of organ size, stress response, and tissue homeostasis; its aberrant hyperactivation is linked to multiple pathological disorders including diabetes. Here we show that MST1 deletion in mice resulted in improved glucose tolerance and insulin secretion, and restored pancreatic β-cell mass as a result of improved β-cell survival and proliferation in the combined high fat/high sucrose and streptozotocin (HFS/STZ) model of β-cell destruction and diabetes. Importantly, the glucose-lowering effects in the MST1-knockout (KO) mice could be accounted to the enhanced β-cell mass and improved insulin secretion without changes in insulin sensitivity. Metabolic and morphological data suggest that normalization of blood glucose and insulin secretion, islet architecture, and β-cell mass by MST1 deletion in response to diabetes-induced injury occurs as a result of improved β-cell survival and proliferation establishing MST1 as potent regulator of physiological β-cell turnover.

## Introduction

Since the discovery of highly activated serine/threonine kinase Mammalian Sterile 20-like kinase 1 (MST1), a core kinase in the Hippo signaling pathway, during pancreatic β-cell failure in the pancreas and diabetes progression [1], MST1 targeted therapies were designed to protect β-cells and prevent diabetic complications, such as nephropathy and cardiomyopathy [2–6].

MST1 acts as a stimulator of caspases to initiate the apoptotic cascade. Reciprocally, caspase-mediated cleavage of MST1 enhances its activity and amplifies a cellular apoptotic response loop establishing a bidirectional interaction between MST1 and the caspase machinery [7, 8]. Such amplification has been observed under chronic conditions of hyperglycemia, so called glucotoxicity, in the pancreatic β-cells, leading directly to β-cell dysfunction with abolished glucose-stimulated insulin secretion (GSIS) through degradation of the important β-cell transcription factor pancreatic duodenal homebox-1 (PDX-1) and ultimately to β-cell failure and death [1]. Also, other stimuli of β-cell failure, such as lipotoxicity and pro-inflammatory cytokines lead to MST1 activation, i.e., its phosphorylation and cleavage and to subsequent caspase loops.

MST1 activation and its downstream events are controlled by a cross-communication between many other signaling pathways of cellular survival and metabolism through non-linear feedback loops to balance cellular turn-over, altogether regulating important biological processes such as tissue development, cellular differentiation, proliferation, stress response, and apoptosis [9–11]. Exemplary, in the β-cell MST1 initiates the intrinsic cell death program through upregulation of the pro-apoptotic BCL-2 member BIM and mitochondria-mediated apoptosis and also interacts with JNK and AKT signaling pathways [1].

Controlled by MST1 and several other kinases, the Hippo pathway has recently been shown to regulate pancreas development, β-cell survival, proliferation, and regeneration [6], as well as stress and metabolic adaptations in metabolically active organs including liver and fat [11] and constitutes an emerging player in both cellular and systemic metabolism [11]. Therefore, it is not surprising, that fluctuations in the Hippo signaling pathway are also involved in the progression of diabetes with its complex and multifactorial metabolic disturbances in glucose homeostasis leading to hyperglycemia. MST1 is not only activated in β-cells under diabetogenic conditions of lipo- and glucotoxicity [1], but also in insulin target tissues, shown in epididymal fat of high fat diet-treated mice [12] as well as in rodent cardiomyocytes [13] and podocytes [14] under high glucose conditions and in the kidney of hyperglycemic IRS2-KO mice [15], indicating its disease-relevant upregulation in several organs during diabetes progression.

Current therapies which restore normoglycemia cannot halt the progressive decline in both insulin secretory function and β-cell mass during deterioration of both type 1 and 2 diabetes (T1D/T2D) [16–20]. There is an urgent need for β-cell directed alternative therapeutic interventions to rebuild highly functional β-cells, that foster β-cell regeneration and/or halt β-cell apoptosis. MST1 is a promising target for such strategy against diabetes. Activated by diabetogenic stimuli in several experimental models of obesity-associated T2D and immune-mediated T1D in vitro and in vivo, MST1 directly triggers β-cell apoptosis and impairs insulin secretion [1]. In this study, we investigated whether in vivo ablation of MST1 would promote β-cell survival and insulin secretion under diabetic conditions in vivo in a severe diabetes model of combined diet-induced obesity and induced β-cell destruction, which resembles both overnutrition and inflammation-induced β-cell stress in T2D as well as T1D.

## Materials and methods

### Animals

**Fig. 1:**
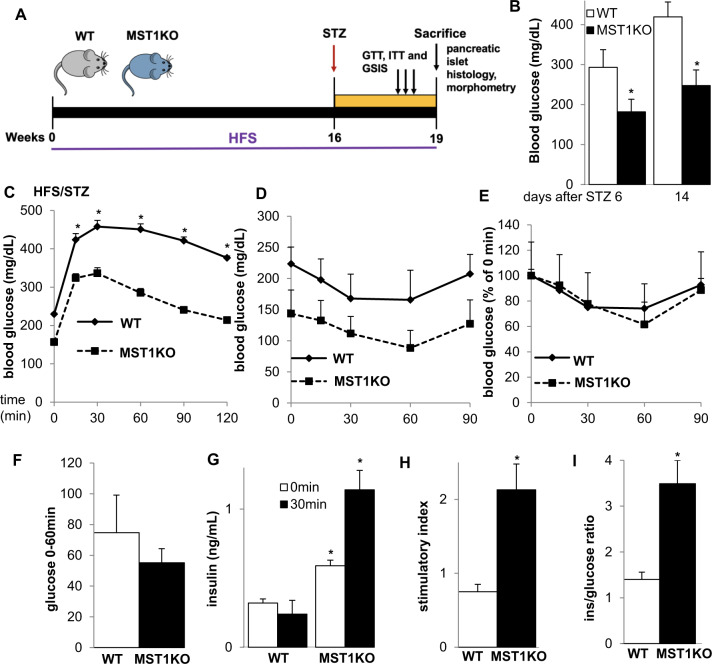
MST1 deletion improves glucose tolerance and insulin secretion in the HFS/STZ-model of diabetes. Whole body MST1KO (*n* = 5) and WT (*n* = 6) mice were fed a high fat/high sucrose diet (HFS) for 16 weeks and thereafter injected with a single dose of STZ (100 mg/kg) and kept for 3 more weeks under HFS treatment. **A** Schematic experimental design. **B** Random blood glucose levels at 6 and 14 days after STZ injection. **C** Intraperitoneal glucose tolerance test (ipGTT) with 1 g/kg BW glucose. Intraperitoneal insulin tolerance test (ipITT) with 0.75IU/kg BW insulin **D** shown in the absolute blood glucose response, **E** normalized to 100% glucose before insulin injection and **F** presented as difference of the highest (0 min) and lowest (60 min) glucose concentration. **G** Insulin secretion during an ipGTT measured before (0 min) and 30 min after glucose injection, **H** data are expressed as ratio of secreted insulin at 30 min/0 min (stimulatory index). **I** The ratio of secreted insulin and glucose is calculated at fed state. **C**–**H** Analyses were performed during week 3 after STZ injection and **I** at the last day of the study. Data are expressed as means ± SEM. *p < 0.05 MST1KO compared to WT littermates.

MST1 knockout (KO) mice kindly provided by Wufan Tao, Fudan University, Shanghai, China) [21] and their wildtype (WT) littermates (age: 8–10 weeks) were fed a high fat/high sucrose diet (HFS, “Surwit” Research Diets, New Brunswick, NJ: 58, 26 and 16% calories from fat, carbohydrate, and protein, respectively [22]) for 16 weeks. Control normal diet (ND) fed WT and MST1KO colonies had been established before and results published [1]. Thereafter, a single dose of 100 mg/kg BW streptozotocin (STZ) was i.p. injected to induce β-cell failure and insulin deficiency and HFS feeding was continued for 3 more weeks. Random blood was obtained from the tail vein of non-fasted mice and glucose measured using a glucometer (Freestyle; Abbott, Alameda, CA). Three weeks after STZ, mice were killed and pancreases dissected (Fig. 1A). All animals were housed in a temperature-controlled room with a 12 h light/dark cycle and were allowed free access to food and water in agreement with the animal care guidelines of the NIH and §8 German animal protection law and approved by the Bremen Senate.

### Intraperitoneal glucose and insulin tolerance tests and measurement of insulin release

Intraperitoneal glucose tolerance tests (ipGTTs) and insulin tolerance tests (ipITTs) were performed as described previously [1]. After 12 h overnight fast, i.p. glucose was injected (40%; B.Braun, Melsungen, Germany; 1 g/kg body weight) and blood glucose measured at time points 0, 15, 30, 60, 90, and 120 min by glucometer FreeStyle. For ipITTs, 0.75 U/kg body weight recombinant human insulin (Novolin, Novo Nordisk) was injected after 5 h fasting and glucose concentrations were determined accordingly. Insulin secretion was measured before (0 min) and after (30 min) i.p. injection of glucose (2 g/kg body weight) by an ultrasensitive mouse insulin Elisa kit (ALPCO Diagnostics, Salem, NH).

### Immunohistochemistry and morphometric analysis

Mouse pancreases were fixed in 4% formaldehyde at 4 °C for 12 h, dehydrated, embedded in paraffin, and processed as described [1]. For proliferation analysis, 4 µm sections were deparaffinized, rehydrated and incubated overnight at 4 °C with anti-Ki67 (M7249, 1:50; Dako) and anti-insulin (A0546, 1:50; Dako) antibodies followed by Cy3/fluorescein isothiocyanate (FITC)-conjugated secondary antibodies (#715-165-150, #706-096-148 Jackson ImmunoResearch Laboratories, West Grove, PA). Slides were mounted with Vectashield with 4′6-diamidino-2-phenylindole (#H-1200-10; DAPI; Vector Labs, USA). β-cell apoptosis for mouse sections was analyzed by the terminal deoxynucleotidyl transferase-mediated dUTP nick-end labeling (TUNEL) technique according to the manufacturer’s instructions (#12156792910 In Situ Cell Death Detection Kit, TMR red; Roche, Mannheim, Germany, now through SigmaI-Aldrich GmbH) and double stained for insulin. Fluorescence and morphometric data were determined by computer-assisted measurements using a Nikon MEA53200 (Nikon GmbH, Dusseldorf, Germany) microscope, and images were acquired using NIS-Elements software (v3.22.11, Nikon) as described [1]. The number of islets (defined as insulin^+^ aggregates of at least 25 µm in diameter) was scored and islet density calculated as a number of islets per cm^2^ of tissue and mean islet size as a ratio of total insulin^+^ area to total islet number. Mean percent β-cell fraction per pancreas was calculated as the ratio of insulin^+^ and whole pancreas section area. For β-cell mass, this fraction was multiplied by the respective mouse pancreas weight. Morphometric β-cell and islet characterizations are results from analyses of at least 100 islets per mouse from sections cut throughout the whole pancreas.

### Statistical analyses

Data are presented as means ± SEM. Mean differences were determined by Student’s t-tests. *P* value <0.05 was considered statistically significant.

## Results and discussion

The combination of HFS for 16 weeks and subsequent administration of a single dose of STZ (100 mg/kg) (Fig. 1A) in a mouse model of harsh diabetes [23] led to severe hyperglycemia at 6 days after administration of STZ and remained throughout the study, together with impaired fasting glucose and glucose intolerance in the WT mice (Fig. 1B, C). In contrast, random glucose levels, as well as glucose tolerance, were significantly improved in the MST1KO mice (Fig. 1B, C).

To assess whether this metabolic improvement was due to changes in insulin sensitivity, we performed an insulin tolerance test. Under the diabetogenic conditions of HFS/STZ, WT and MST1KO mice had a similar response to exogenous insulin (Fig. 1D–F), especially seen when fasted glucose levels were both normalized to 100% (Fig. 1E), suggesting that the glucose-lowering effects in the MST1KO mice cannot be accounted to a systemic improvement in insulin sensitivity. The major role of pancreatic β-cells is to regulate the production of insulin in response to fluctuations in blood glucose levels. To illustrate the possible impact of MST1 on the β-cell insulin secretory response under diabetogenic HFS/STZ treatment, we performed in vivo GSIS measurements. Glucose-induced insulin secretion was fully blunted in the HFS/STZ-treated WT mice, but robustly restored in MST1KO mice; a significant increase in both basal (0 min) and glucose-stimulated insulin levels (30 min) was found in HFS/STZ treated MST1KO mice compared to WT counterparts (Fig. 1G, H), indicating a higher insulin secretion per se by MST1 deleted islets (Fig. 1G) as well as a 2.5-fold improved GSIS (Fig. 1H). Also, a robust elevation in insulin-to-glucose ratio was observed in MST1KO mice, compared to WT controls (Fig. 1I).

**Fig. 2:**
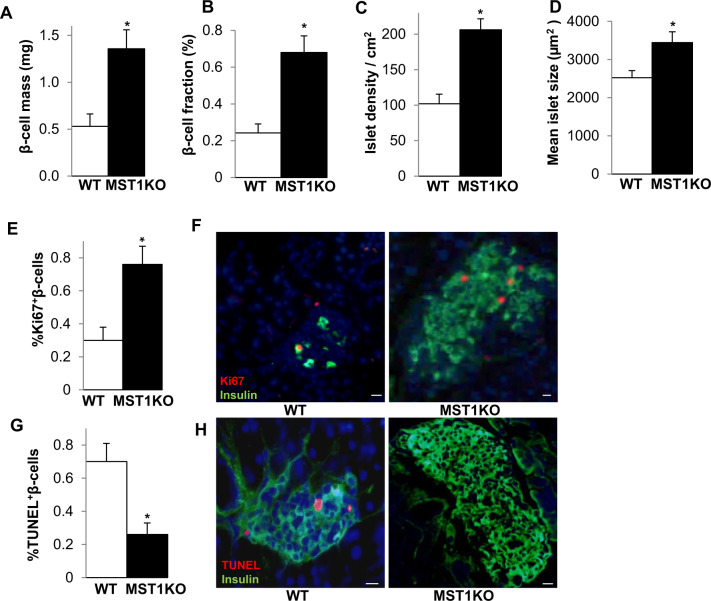
MST1 deletion protects pancreatic β-cells in HFS/STZ-model of diabetes. Whole body MST1KO (*n* = 5) and WT (*n* = 6) mice were fed a high fat/ high sucrose diet (HFS) for 16 weeks and thereafter injected with a single dose of STZ (100 mg/kg) and kept for 3 more weeks under HFS treatment. Fixed, paraffin-embedded mouse pancreas sections spanning the width of the pancreas were stained for insulin and **A** β-cell mass as cross-sectional area of insulin-positive cells/whole pancreas × pancreas mass in mg, **B** percentage of β-cell fraction of the whole pancreas **C** islet density/cm^2^ pancreas and **D** mean islet size was analyzed using NIS-elements microscopical analysis software. Quantitative analyses (**E**, **G**) and representative images (**F**, **H**) from triple staining for Ki67 (**E**, **F**) or TUNEL (**G**, **H**), insulin, and DAPI expressed as a percentage of TUNEL- or Ki67-positive β-cells ±SEM. Data are expressed as means ± SEM. *p < 0.05 MST1KO compared to WT littermates. Scale bars depict 20 μm.

Consistent with the improved metabolic phenotype in the HFS/STZ model, histological examination of the pancreas and quantification of β-cell mass revealed a marked induction in β-cell mass in MST1KO mice compared to WT controls (Fig. 2A). Consistent with the β-cell mass, β-cell volume, islet density, and islet size all were significantly restored in MST1KO, compared to HFS/STZ-WT mice (Fig. 2B–D). These data are in line with our previous report on the protective effect of MST1-deletion during high fat/high sucrose induced hyperglycemia and β-cell failure [1]. The most recognized functional output of MST1 is to regulate cell survival and proliferation. Cell cultures as well as in vivo mouse models showed that loss-of-function of MST1 and its downstream targets leads to increased proliferation, and confers a resistance to apoptosis leading to tissue growth and regeneration in multiple tissues supporting the functional role of Hippo as strong regulator of organ size [24–31]. To investigate whether the restoration in β-cell mass and islet size and density was a result of elevated β-cell numbers due to increased β-cell proliferation and/or diminished β-cell apoptosis, we next assessed β-cell proliferation by Ki67 and apoptosis by TUNEL in response to HFS/STZ treatment. A significant increase in Ki67/insulin-co-positive β-cells (Fig. 2E, F) together with a markedly reduced TUNEL/insulin-co-positive β-cells was observed in HFS/STZ-treated MST1KO, compared to the WT control group (Fig. 2G, H).

This suggests a combined additive impact on augmented proliferation as well as reduced apoptosis as mechanism of β-cell mass restoration in MST1KO mice during STZ-induced β-cell destruction under metabolic pressure triggered by HFS feeding. This is in line with our previous observations that the loss of MST1 fosters compensatory hyperplasia and restoration of β-cell function in other less severe preclinical models of diabetes [1]. Our data confirm that MST1 is highly activated in β-cells under diverse diabetogenic conditions, as shown before in isolated islets from diabetic mice as well as from patients with T2D suggesting its activation as an integral event in the process of β-cell failure in diabetes [1].

In this study, we explicitly used general MST1KO mice in order to assess the main target tissue for the protective effect of MST1 deletion in the harsh combined model of initial HFS-induced impairment in insulin sensitivity and additional STZ-induced potentiation of β-cell failure. Shown by insulin tolerance tests, insulin sensitivity remained unchanged by MST1-deletion, while β-cell function, mass, proliferation, and survival were well-improved.

Through deletion of the MST1-mediated death signal, we have uncovered an important deleterious action of MST1 to mediate β-cell dysfunction, apoptosis, and impaired proliferation in response to diabetic injuries. MST1 deletion did not only prevent HFS/STZ-induced β-cell death, but also improved the capacity of the β-cell to produce insulin and thus restored a fully functional β-cell mass as depicted by a truly physiological glucose tolerance.

Altogether, our data show, that MST1-deleted β-cells can cope with the complex milieu to maintain a compensatory stress response. MST1 ablation normalized glucose tolerance, improved insulin secretion and β-cell mass in the HFS/STZ mouse model of β-cell destruction and diabetes. Given that the hyperactivity of the Hippo kinase MST1 is linked with β-cell apoptosis and the development of diabetes, small-molecule inhibitors that inhibit its kinase activity could restore β-cell survival and insulin secretion. In that regard, we have recently shown that neratinib, a previously unrecognized inhibitor of MST1, represents a promising β-cell-protective drug with robust proof-of-concept in vitro in human islets and in vivo in rodent models of both T1D and T2D [2].
